# Sortilin Participates in Light-dependent Photoreceptor Degeneration in Vivo

**DOI:** 10.1371/journal.pone.0036243

**Published:** 2012-04-27

**Authors:** Ana M. Santos, Noelia López-Sánchez, David Martín-Oliva, Pedro de la Villa, Miguel A. Cuadros, José M. Frade

**Affiliations:** 1 Department of Cell Biology, University of Granada, Granada, Spain; 2 Cajal Institute, IC-CSIC, Madrid, Spain; 3 Department of Physiology, School of Medicine, University of Alcalá, Alcalá de Henares, Madrid, Spain; University of Florida, United States of America

## Abstract

Both proNGF and the neurotrophin receptor p75 (p75^NTR^) are known to regulate photoreceptor cell death caused by exposure of albino mice to intense illumination. ProNGF-induced apoptosis requires the participation of sortilin as a necessary p75^NTR^ co-receptor, suggesting that sortilin may participate in the photoreceptor degeneration triggered by intense lighting. We report here that light-exposed albino mice showed sortilin, p75^NTR^, and proNGF expression in the outer nuclear layer, the retinal layer where photoreceptor cell bodies are located. In addition, cone progenitor-derived 661W cells subjected to intense illumination expressed sortilin and p75^NTR^ and released proNGF into the culture medium. Pharmacological blockade of sortilin with either neurotensin or the “pro” domain of proNGF (pro-peptide) favored the survival of 661W cells subjected to intense light. In vivo, the pro-peptide attenuated retinal cell death in light-exposed albino mice. We propose that an auto/paracrine proapoptotic mechanism based on the interaction of proNGF with the receptor complex p75^NTR^/sortilin participates in intense light-dependent photoreceptor cell death. We therefore propose sortilin as a putative target for intervention in hereditary retinal dystrophies.

## Introduction

Neurotrophins are a family of proteins that include nerve growth factor (NGF), brain derived neurotrophin factor, neurotrophin-3, and neurotrophin-4/5, which are known to regulate the development and maintenance of the nervous system [1]. These proteins transduce their signals through two different transmembrane receptors: Trk receptor tyrosine kinases [2] and the p75 neurotrophin receptor (p75^NTR^) [3]. The latter is a member of the NGF/tumor necrosis factor (TNF) receptor superfamily, which has been shown to cooperate with Trks to induce survival and differentiation [2]. p75^NTR^ can also induce proapoptotic signals [4], which are initiated in vivo by immature proneurotrophin forms [5]–[7], including proNGF. Indeed, proNGF is the predominant form of NGF in the brain [8] and has been shown to induce apoptosis in different neurodegenerative conditions [8]–[10].

High-affinity binding of proNGF to p75^NTR^ appears to be mediated by the interaction of the “pro” domain of the former (pro-peptide) with sortilin [11], [12], a transmembrane receptor containing a Vps10p domain [13]. Sortilin would act as a necessary co-receptor of p75^NTR^ to promote apoptosis in different cell systems, including the developing retina [11], [14], [15]. In fact, the expression of both p75^NTR^ and sortilin is increased after neuronal stress situations such as facial nerve injury [16] or retrovirus-induced spongiform encephalomyelopathy [17]. Moreover, the proNGF/sortilin/p75^NTR^ complex has been shown to participate in neurodegenerative processes, including Parkinson's disease [18] and age-related neurodegeneration [14], [19], [20].

Retinitis pigmentosa (RP) is a heterogeneous group of hereditary retinal dystrophies characterized by progressive photoreceptor degeneration of apoptotic nature, due to mutations affecting to basic rod physiology [21], [22]. RP initially manifests as night blindness with peripheral visual field loss and frequently results in full visual loss. Several animal models of RP are currently available, including a number of mouse and rat mutants [23]. Photoreceptor degeneration reminiscent of RP is also observed in albino mice after chronic exposure to moderate illumination or acute exposure to intense illumination [24]. Upregulation of p75^NTR^ expression has been demonstrated in the retina of light-exposed albino mice [25]–[27] and has also been reported in the cone-progenitor-derived cell line 661W after acute illumination with intense light [27]. p75^NTR^ is expressed by the human retina [28] and has been reported to participate in photoreceptor degeneration driven by intense illumination in Wistar rats and p75^NTR^ knock-out mice [25], as well as in 661W cells [27]. Interestingly, lack of p75^NTR^ expression does not protect photoreceptors from light-induced cell death in *Ngfr* ^−/−^ albino mice [29], suggesting that other pathways may trigger the apoptotic signal in these mice. In contrast, the loss of one copy of the gene encoding p75^NTR^ in *Ngfr* ^+/−^ albino mice provided significant neuroprotection upon constant lighting [29]. Increased expression of proNGF has been reported in the retina of RCS rats [30], and proNGF is known to induce apoptosis in 661W cells [30]. Despite these data on the participation of proNGF and p75^NTR^ in photoreceptor degeneration, the involvement of sortilin in this process has yet to be demonstrated. We therefore hypothesized that sortilin plays a role in RP models in which proNGF/p75^NTR^ is known to trigger apoptosis.

In this study, we present evidence that sortilin and p75^NTR^ are both expressed in photoreceptors exposed to intense light, and that these cells also produce proNGF. Pharmacological inhibition of sortilin partially prevents the death of 661W cells as well as of photoreceptors in vivo. Hence, sortilin may be useful for pharmacological intervention in hereditary retinal dystrophies. This would avoid targeting p75^NTR^, which also participates in other, sometimes beneficial, neural processes [3].

## Results

### Intense light enhances p75^NTR^ Expression within the Outer Nuclear Layer (ONL) in Vivo

Several observations suggest that p75^NTR^ is involved in photic injury. In this regard, p75^NTR^ expression has been shown to correlate with intense light-dependent photoreceptor loss [25], [27], while enhanced survival of photoreceptors can be observed in *Ngfr* ^+/−^ albino mice maintained under constant light [29]. In order to confirm that the mRNA encoding p75^NTR^ can be expressed by the photoreceptors in response to intense light, we performed in situ hybridization with *Ngfr* specific probes in retinal cryosections from albino mice subjected to intense illumination and from control mice. The illumination procedure used for this study was previously shown to result in photoreceptor degeneration, which was already observed at 6 h after lighting [31]. The diverse neuronal types present in the adult retina are disposed within three layers: the ganglion cell layer (GCL), which contains the somas of retinal ganglion cells (RGCs) and displaced amacrine cells; the inner nuclear layer (INL), which is constituted by the somas of bipolar, horizontal, amacrine, and Müller cells and displaced RGCs; and the ONL, which contains the somas of the photoreceptors. Therefore, it is possible to determine whether a particular gene is expressed by particular neural types by using simple morphological criteria.

**Figure 1:**
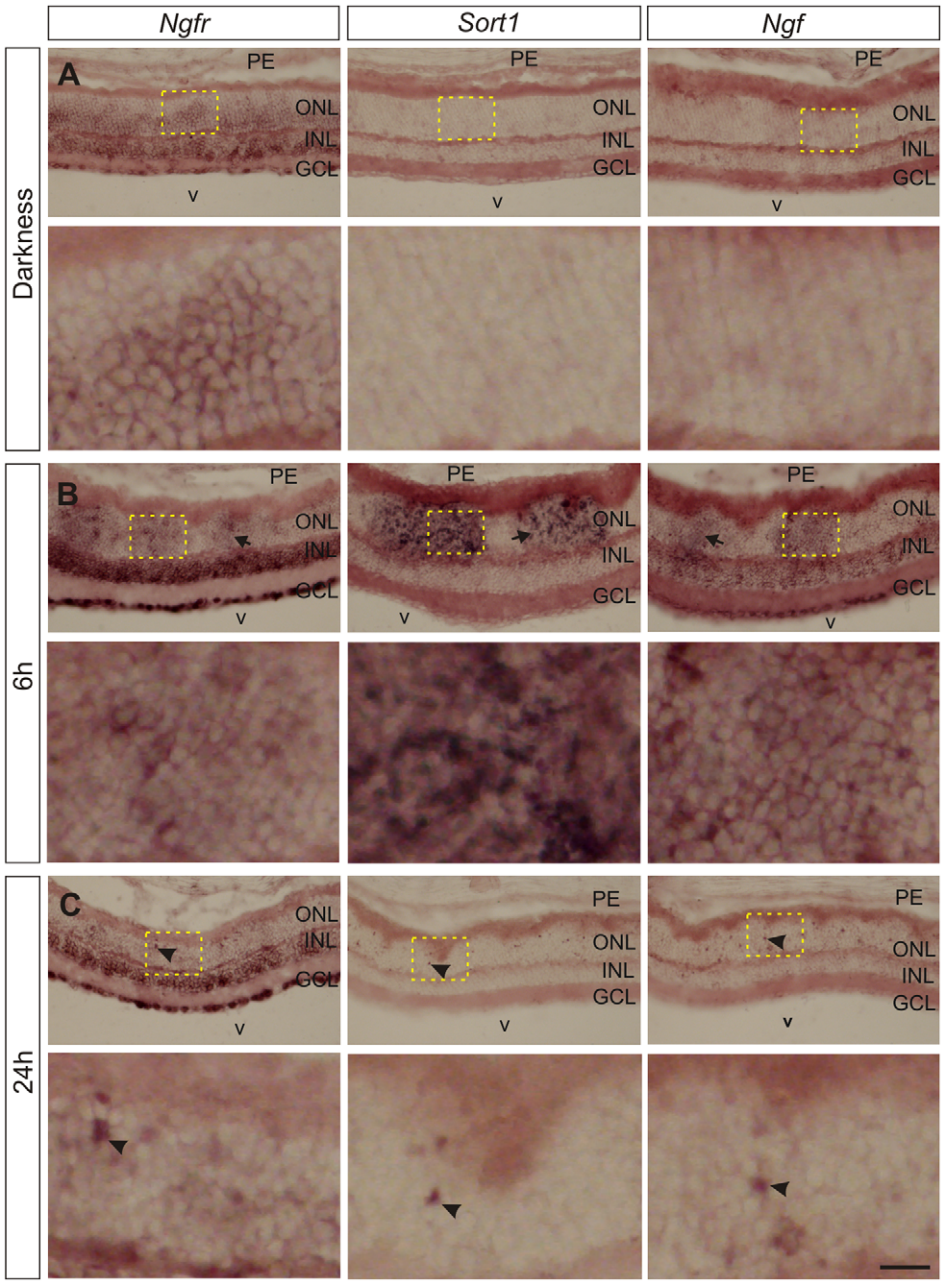
Expression of *Ngfr*, *Sort1* and *Ngf*, determined by in situ hybridization, in normal and intense light-treated retinas. Cryostat sections of the retina of BALB/c mice (n = 3 per experimental point) maintained in darkness (A) or subjected to intense light for 7 h and then kept in darkness for 6 (B) or 24 h (C) were processed for in situ hybridization with *Ngfr* (left column), *Sort1* (middle column), or *Ngf* (right column) probes. Specific labeling appears in dark brown. Representative images are shown. (B) Particular regions of the outer nuclear layer (ONL) were enriched in these mRNAs 6 h after illumination. Observe that the staining is not uniform across the ONL and that some patches show the most intense labeling (arrows). (C) Twenty-four h after light treatment, a clear decrease of the expression of all three transcripts is seen in the ONL, although some cells, unevenly distributed throughout this layer (arrowheads), continue to show intense expression. V: vitreous body, PE: pigment epithelium, GCL: ganglion cell layer, INL: inner nuclear layer. Lower panels represent higher magnification of boxed areas in upper panels. Bar: 100 µm (upper panels); 500 µm (lower panels).

In the control, non-illuminated mice, *Ngfr-*specific mRNA was detected in all retinal layers, including the ONL (Fig. 1A, left panels). This pattern was modified by intense light treatment. Thus, enhanced *Ngfr* expression was detected at 6 h after lighting in both the GCL and INL, whereas in the ONL *Ngfr* expression remained at similar levels as in the control (Fig. 1B, left panels). At 24 h post-illumination, when strong cell death in the ONL can still be observed [31], *Ngfr-*specific mRNA remained at high levels in the GCL and INL but only a few scattered cells from the ONL showed increased *Ngfr* expression (Fig. 1C, left panels). Hence, photoreceptors appear to constitutively express *Ngfr* in albino mice, and intense lighting enhances its expression in specific regions following a dynamic spatiotemporal pattern.

**Figure 2:**
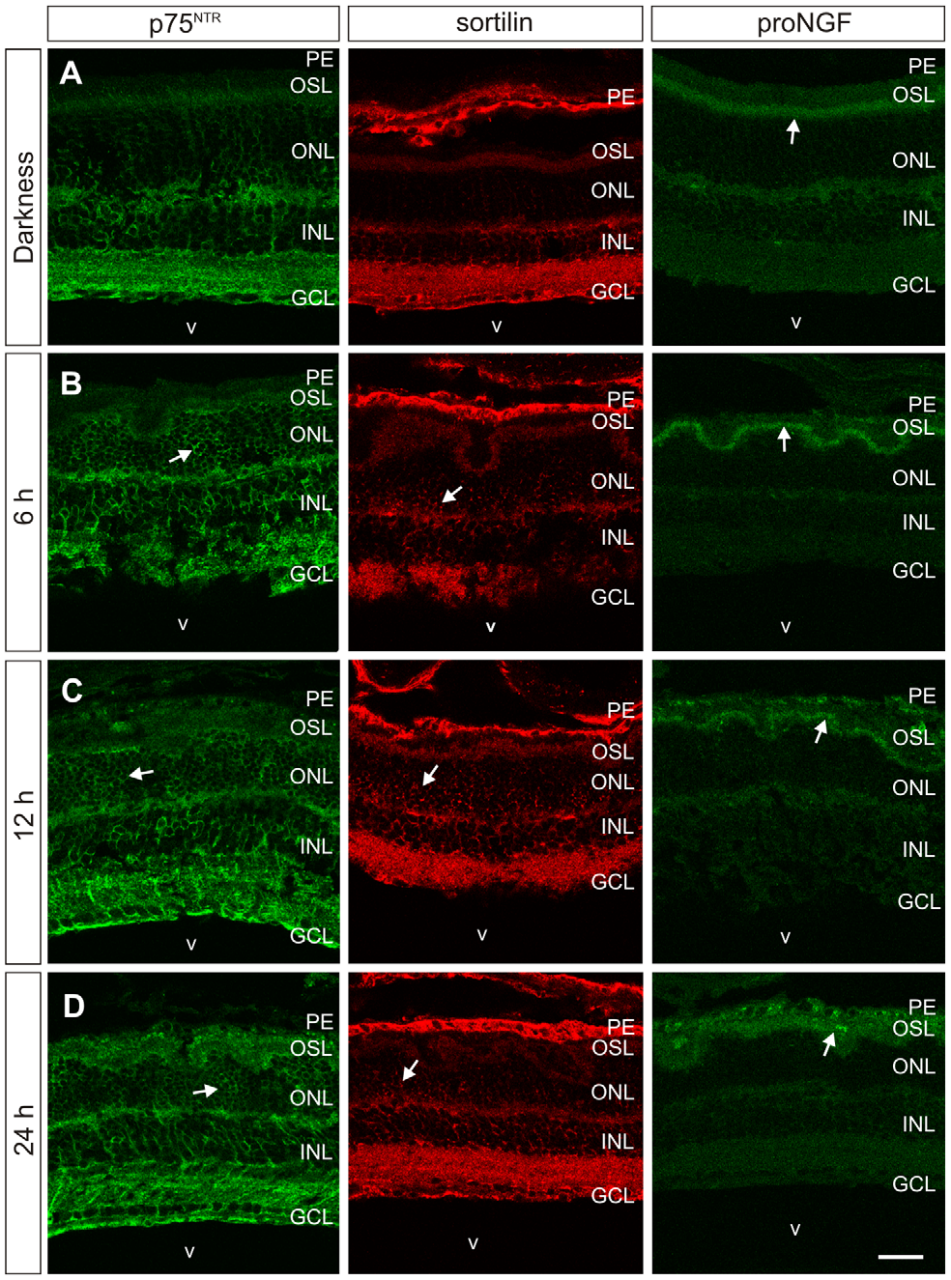
Intense light induces the expression of p75^NTR^, sortilin, and proNGF proteins in the ONL. Cryostat sections of the retina of BALB/c mice (n = 3 per experimental point) maintained in darkness (A) or subjected to intense light for 7 h and then kept in darkness for 6 (B), 12 (C), or 24 h (D) were immunolabeled with specific antibodies for p75^NTR^ (green, left column), sortilin (red, middle column), or proNGF (green, right column). Representative images are shown. (A) Untreated retinas showed p75^NTR^ immunostaining (left panel) in nearly all layers, although the labeling was weak in the outer nuclear layer (ONL). The sortilin antibody bonded mainly to the ganglion cell layer (GCL) and inner plexiform layer but was virtually absent from the ONL (A, middle panel). Finally, the proNGF antibody showed an immunoreactive band at the level of photoreceptor outer segments (OSL). (B) Six hours after light treatment, the intensity of the immunolabeling was increased in all three cases. Most ONL cells were strongly labeled with p75^NTR^ antibody, although the predominant expression of p75^NTR^ was detected in some patches within this layer (left panel). Sortilin immunostaining (middle panel) was also increased within the ONL, although restricted to particular areas. Finally, proNGF antibody (right panel) showed a similar distribution pattern to that described in untreated retinas, though the immunopositive band at the OSL was more intense. Similar patterns of immunoreactivity were seen at 12 h (C) and 24 h (D) after light treatment. Arrows indicate areas showing specific immunostaining within the ONL and OSL. V: vitreous body, PE: pigment epithelium, INL: inner nuclear layer. Bar: 50 µm.

In order to verify that not only the transcripts but also p75^NTR^ protein is expressed in the retina of albino mice subjected to intense light, retinal cryosections were immunostained with an anti-p75^NTR^ specific antibody. As expected from the generalized expression of the *Ngfr* transcript in the control retina, p75^NTR^ was detected in all retinal layers in mice adapted to darkness (Fig. 2A, left panel). Intense light induced prominent p75^NTR^ protein expression in groups of cells within the ONL, although low levels of p75^NTR^ remained in most cells located in this retinal layer. This pattern was already detected at 6 h post-illumination and persisted throughout the study period (Fig. 2B–D, left panels). At 24 h post-illumination, high levels of p75^NTR^ protein continued to be detected in the ONL, where only a few cells showed strong *Ngfr* expression (see Fig. 1C, left panels), suggesting that the half life of p75^NTR^ is longer than that of the *Ngfr* transcript.

### Intense Light Transiently Induces Sortilin Expression within the ONL in Vivo

Proapoptotic signals triggered by proNGF are known to require the presence of the receptor sortilin for inducing their effect [11]. In order to determine whether mRNA encoding sortilin is expressed in cells within the ONL in response to intense light, we performed in situ hybridization with a *Sort1* specific probe in both control mice and mice subjected to intense lighting. In control mice, mRNA for *Sort1* was not expressed at detectable levels in any retinal layer (Fig. 1A, middle panels). In contrast, intense lighting induced a marked burst of *Sort1* expression in patches located within the ONL, which was already observed at 6 h post-illumination (Fig. 1B, middle panels), suggesting that intense light does not homogeneously affect all photoreceptors. Twenty four hours later, only a few cells randomly scattered throughout the ONL showed elevated *Sort1* expression (Fig. 1C, middle panels), similar to observations for *Ngfr* (Fig. 1C, left panels).

Sortilin protein was detected by immunostaining, using a specific anti-sortilin antibody [14], in the retinas of control mice and mice subjected to intense light. In control retinas, sortilin was detected in the GCL and inner plexiform layer but not in the ONL (Fig. 2A, middle panel), suggesting that low mRNA expression levels of *Sort1*, undetectable by in situ hybridization, could account for this expression pattern. In contrast, conspicuous sortilin expression appeared at 6 h after illumination within the ONL, showing stronger intensity in certain areas (Fig. 2B–D, middle panels). As described above for p75^NTR^, ONL labeling with the sortilin antibody still appeared at 24 h after illumination (Fig. 2D, middle panel) but only a few cells showed strong *Sort1* mRNA expression (Fig. 1C, middle panels). This suggests that the half life of the protein is longer than that of the *Sort1* mRNA, and that sortilin remains in the retina despite the fall in mRNA expression.

As nearly all cells in the ONL show some degree of p75^NTR^ labeling (see the former section), it was apparent that those cells expressing sortilin can also express the 75^NTR^ co-receptor.

### Intense Light Transiently Induces proNGF Expression within the ONL in Vivo

ProNGF can induce apoptosis through the p75^NTR^/sortilin complex. In order to determine whether proNGF is expressed in the ONL in response to intense lighting, in situ hybridization was performed using an *Ngf* specific probe in retinal cryosections from control mice and mice subjected to intense light. *Ngf* expression was undetectable in control retinas (Fig. 1A, right panels) but was upregulated in cells within the GCL and INL and in patches within the ONL at 6 h post-illumination (Fig. 1B, right panels). As occurs with *Sort1*, *Ngf* expression in the retina was virtually absent at 24 h post-illumination, except in a few unevenly distributed cells within the ONL (Fig. 1C, right panels).

In order to verify whether proNGF protein is expressed in the retina of mice subjected to intense illumination, retinal cryosections were immunostained with an anti-proNGF antibody that specifically recognizes the pro-domain [32], thereby excluding the mature form of NGF from the analysis. Low levels of proNGF protein were observed in the photoreceptor outer segment layer (OSL) of control mice (Fig. 2A, right panel). This same pattern was maintained after intense illumination, although the intensity of labeling increased in the OSL and did not decrease at later time points (Fig. 2B–D, right panels). Therefore, proNGF appears to be stored/released in the region encompassing the photoreceptor outer segments and subretinal space. In contrast to the observation that intense light induces *Ngf* expression in the GCL and INL (see Fig. 1B,C), proNGF protein could not be detected in these layers, suggesting that translation of the *Ngf* transcript is prevented in these retinal layers and/or that the mature form of NGF, also encoded by the *Ngf*-specific mRNA, is preponderant in the GCL and INL. In addition, proNGF expressed by the RGCs may be transported to retinal target tissues.

**Figure 3:**
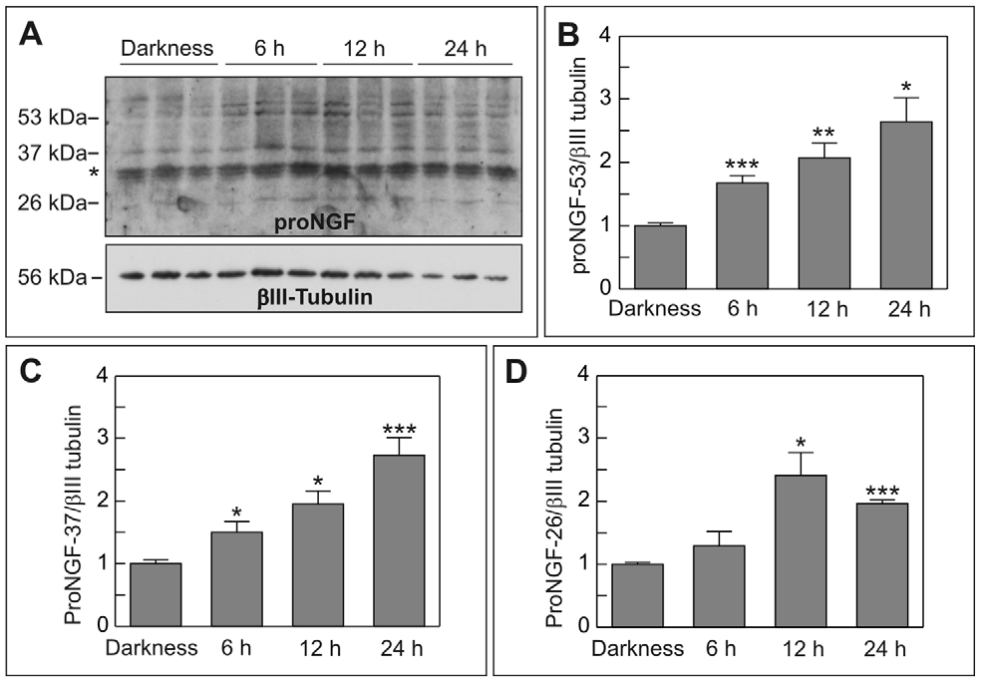
Expression of proNGF in the retina of albino mice subjected to intense illumination. (A) Total retinal extracts from BALB/c mice subjected to intense light for 7 h and then kept in darkness for the indicated time points (6, 12 and 24 h) or those maintained in darkness without previous lighting (Darkness), were subjected to western blot analysis using either an antibody specifically recognizing the pro domain of proNGF (upper panel) or an anti-βIII tubulin antibody (bottom panel in A). This analysis revealed the presence in the extracts of the previously described forms of proNGF with apparent molecular weights of 53, 37, and 26 kDa. Each lane represents a different retinal extract (n = 3 per treatment). Asterisk marks an unspecific band. (B) Quantitative variations of the proNGF form of 53 kDa, normalized to βIII tubulin (mean ± SEM). (C) Quantitative variations of the proNGF form of 37 kDa, normalized to βIII tubulin (mean ± SEM). (D) Quantitative variations of the proNGF form of 26 kDa, normalized to βIII tubulin (mean ± SEM). *p<0.05; **p<0.01; ***p<0.005 (n = 3; Student's t test).

Unlike p75^NTR^ and sortilin, proNGF expression was restricted to a narrow retinal layer, allowing the use of total retinal extracts to confirm the increase in proNGF post-illumination without contamination from the INL and GCL. Western blot analysis confirmed the presence in control retinas of specific proNGF bands previously described in the human brain [32], with apparent molecular weights of 53, 37, and 26 kDa (Fig. 3A). Expression of these bands, normalized to βIII tubulin, progressively increased with longer post-illumination time (Fig. 3B–D).

### 661W Cells Express p75^NTR^, Sortilin and ProNGF in Response to Intense Light

The above results suggest an auto/paracrine scenario for the participation of proNGF in photoreceptor cell death. In response to lighting, photoreceptors would produce and release proNGF, which would interact with the proNGF sortilin/p75^NTR^ receptor complex that is also expressed by these cells, thereby facilitating their death. The participation of proNGF and p75^NTR^/sortilin in intense light-induced photoreceptor degeneration was further analyzed by using the cone progenitor-derived 661W cell line as a model system. These cells are optimal for this analysis because they die in response to proNGF [30] and they are killed under intense illumination *via* a p75^NTR^-dependent mechanism [27]. We hypothesized that, in accordance with the in vivo data, intense light would increase the expression of proNGF by 661W cells, which would then be released into the culture medium and trigger p75^NTR^/sortilin-dependent apoptosis.

**Figure 4:**
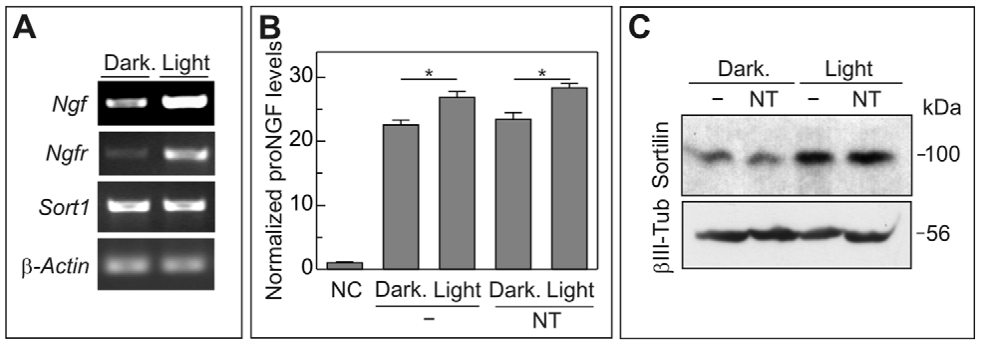
Expression of proNGF, sortilin, and p75^NTR^ in 661W cells subjected to intense light. (A) Cultures of 661W cells grown to 80% confluence were either illuminated for 3 h (Light) or maintained in darkness (Dark.) cDNAs prepared from these cells 2 h post-illumination were amplified with primers specific for *Ngf*, *Ngfr*, *Sort1*, or *β-actin*. RT-PCR from control cDNAs lacking reverse transcriptase did not show specific amplification (not shown). Note that all of these genes are expressed by the 661W cells, and lighting increases the expression of *Ngf* and *Ngfr*. (B) 661W cells grown to 80% confluence were cultured in the presence (NT) or absence (-) of 10 µM neurotensin and illuminated for 3 h (Light) or maintained in darkness (Dark.). Conditioned media collected 24 h after treatment were subjected to ELISA using antibodies recognizing the mature form of NGF and the pro-peptide of proNGF. The levels of released proNGF, normalized to non-conditioned (NC) medium, are shown. Note that proNGF is constitutively produced by the 661W cells and that lighting induces a significant increase in the levels of released proNGF. *p<0.05 (n = 3; Student's t test). (C) 661W cells grown to 80% confluence were illuminated for 3 h (Light) or maintained in darkness (Dark.) in the presence (NT) or absence (-) of 10 µM neurotensin. Total extracts from 661W cells at 3 h after treatment were studied by western blot using antibodies specific for sortilin (Sortilin) or βIII tubulin (βIII-Tub). A representative western blot shows that the amount of sortilin protein is considerably increased after light treatment and that the presence of neurotensin does not influence this increase.

The presence of the *Ngf*, *Ngfr*, and *Sort1* specific mRNAs was analyzed in 661W cells subjected to intense illumination for 3 h or left untreated [27]. Two hours later, the mRNA from these cells was extracted for reverse transcriptase polymerase chain reaction (RT-PCR) analysis using specific primers, demonstrating that 661W cells constitutively express *Ngf*, *Ngfr* and *Sort1*. Lighting enhanced the expression of *Ngf* and *Ngfr*, whereas *Sort1* expression was similar to that in controls (Fig. 4A). The increase in *Ngfr* after lighting is consistent with a previous report of enhanced p75^NTR^ expression in illuminated 661W cells [27]. The release by 661W cells of proNGF into the culture medium was analyzed by enzyme-linked immunosorbent assay (ELISA), using the anti-NGF mAb27/21, known to bind to the native form of mature NGF [33], and an antiserum recognizing the pro-peptide. Control 661W cells and those subjected to intense light for 3 h were incubated for a further 24 h, and the respective culture media were used to measure relative levels of proNGF. This analysis demonstrated that 661W cells release proNGF to the culture medium even under control conditions, showing a significant increase in ELISA signal in comparison to unconditioned media (Fig. 4B). Interestingly, 3 h of treatment with intense light produced a statistically significant increase in proNGF levels in the culture medium (Fig. 4B), consistent with the observed increase in *Ngf* mRNA levels (see Fig. 4A). Finally, western blot analysis demonstrated that sortilin protein was increased in extracts from 661W cells subjected to intense light (Fig. 4C), suggesting its participation in cell death. This increase in sortilin levels is likely to derive from post-transcriptional mechanisms, given that *Sort1* mRNA levels do not change in response to lighting (Fig. 4A).

Overall, these results demonstrate that 661W cells subjected to intense light express p75^NTR^ and sortilin and release proNGF into the culture medium. Therefore, proNGF signaling *via* the p75^NTR^/sortilin complex may be involved in the cell death of 661W cells induced by intense illumination.

### Inhibition of Sortilin Prevents Degeneration of 661W Cells

**Figure 5:**
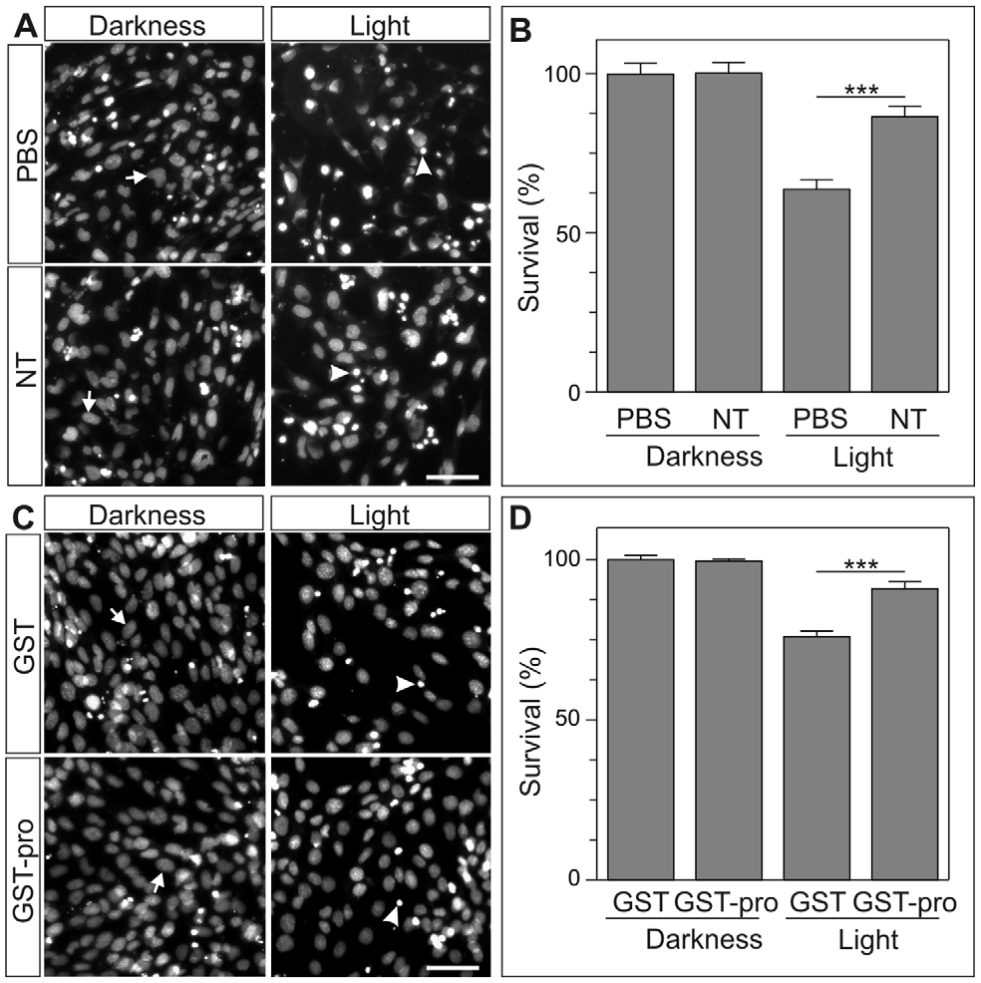
Sortilin inhibition partially prevents intense light-dependent degeneration of 661W cells. 661W cells grown to 80% confluence were illuminated for 3 h (Light) or maintained in darkness (Darkness) in the presence (NT) or absence (PBS) of 10 µM neurotensin (A,B) or in the presence of 100 nM GST-pro (GST-pro) or 100 nM GST (GST) (C,D). Cultures were then returned to the incubator and cultured for 24 h. Finally, cell cultures were fixed and their nuclei were labeled with bisbenzimide to identify pyknotic nuclei. (A) Representative images of nuclei from 661W cells subjected to the indicated treatments. Arrows: non pyknotic nuclei; arrowheads: pyknotic nuclei. (B) Quantification of 661W cell survival under the conditions described in A, as defined by the percentage of cells lacking pyknotic nuclei. (C) Representative images of nuclei from 661W cells subjected to the indicated treatments. Arrows: non-pyknotic nuclei; arrowheads: pyknotic nuclei. D. Quantification of 661W cell survival under the conditions described in C, as defined by the percentage of non- pyknotic nuclei. ***p<0.005 (Student's t test) with respect to PBS or GST light-exposed cultures. Bar: 50 µm.

In order to test the hypothesis that proNGF participates in light-dependent degeneration of 661W cells by activating the p75^NTR^/sortilin complex, we prevented sortilin function in 661W cell cultures by means of two known blocking agents. We first employed neurotensin, a known sortilin ligand that competes with proNGF for sortilin binding and is known to prevent p75^NTR^/sortilin-dependent apoptosis when used at 10 µM concentration [11], [20]. 661W cells cultured in the presence or absence of 10 µM neurotensin were subjected to intense light for 3 h and then maintained in darkness for 24 h. Intense light reduced the survival of 661W cells, as defined by the number of non-pyknotic nuclei remaining in the culture (Fig. 5A,B). The presence of 10 µM neurotensin significantly increased the proportion of cells containing non-pyknotic nuclei (Fig. 5A,B) but did not affect proNGF release (Fig. 4B) or sortilin expression (Fig. 4C).

All of these observations suggest that proNGF, acting through sortilin, participates in the induction of cell death in 661W cells subjected to lighting. To confirm this result, 661W cells were treated with glutathione S-transferase (GST)-pro, a fusion protein containing a peptide mimicking the “pro” domain of proNGF and GST [11]. This chimeric protein has been shown to block sortilin-dependent cell death [11], [34]. 661W cells cultured in the presence of either 100 nM GST or 100 nM GST-pro were subjected to intense light for 3 h and then maintained in darkness for 24 h. The survival of 661W cells was higher in the presence of the GST-pro fusion protein than in the presence of the GST (Fig. 5C,D). Overall, these results indicate that endogenous proNGF released to the culture medium facilitates the death of 661W cells subjected to intense illumination, likely due to the known light-dependent increase in their p75^NTR^ expression at both mRNA (Fig. 4A) and protein level [27], and the observed increase of sortilin expression (Fig. 4C).

### Inhibition of sortilin with GST-pro reduces light-dependent photoreceptor degeneration in vivo

The participation of sortilin in photoreceptor degeneration in vivo was tested in albino mice exposed to intense light [31] and then intraocularly injected with sortilin blockers. After 24 h in darkness, mice were sacrificed and cell death was measured in retinal extracts as the amount of nucleosomes present in the cytosol, as previously described [4], [31]. Considering the volume of vitreous humor in the mouse to be around 7 µl [35], the amount of sortilin blockers injected was adjusted to obtain a similar final concentration to that which shows inhibitory effects in vitro. Hence, the experimental eye was injected with 1 µl of either 18 µM neurotensin or 1.5 µM GST-pro, whereas the contralateral (control) eye was injected with 1 µl of either phosphate buffered saline (PBS) or 1.5 µM GST, respectively.

**Figure 6:**
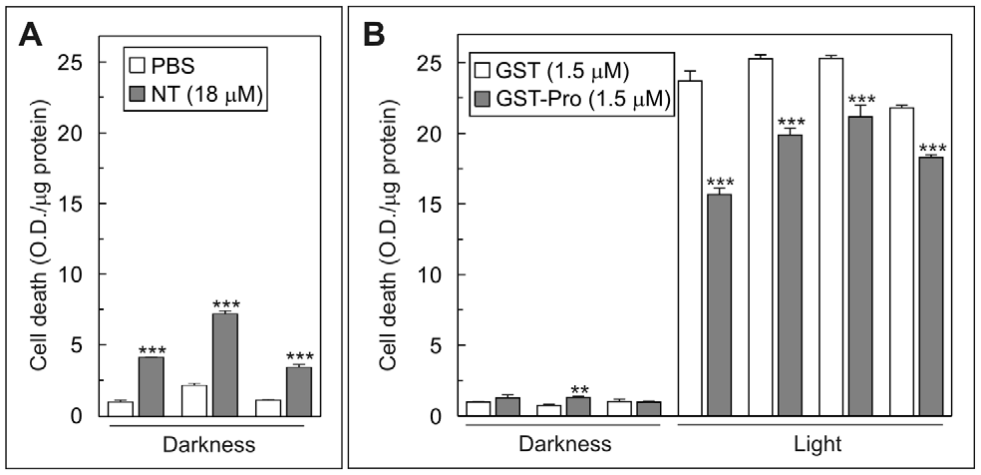
Sortilin inhibition reduces intense light-dependent degeneration in vivo. (A) Eyes from albino mice (n = 3) maintained in darkness (Darkness) were injected with 1 µl of either PBS (experimental eye, white bars) or 90 µM neurotensin (contralateral eye, grey bars). Cell death was measured by ELISA 24 h later as a function of the levels of soluble nucleosomes present in cytoplasmic extracts with respect to the total amount of protein (n = 3) (arbitrary units). The observed increase in soluble nucleosomes indicated that neurotensin have toxic activity. (B) Eyes from albino mice maintained in darkness (Darkness, n = 3) or subjected to intense light for 7 h (Light, n = 4) were injected with 1 µl of either 1.5 µM GST (experimental eye, white bars) or 1.5 µM GST-pro (contralateral eye, grey bars). Cell death was measured by ELISA (in triplicate) 24 h later as a function of the level of soluble nucleosomes present in cytoplasmic extracts with respect to the total amount of protein (arbitrary units). **p<0.01, ***p<0.005 (Student's t test).

The toxicity of the sortilin blockers at these concentrations was tested by injecting them into the eye of non-illuminated albino mice, finding a significant increase in apoptosis with neurotensin versus PBS at 24 h after injection (Fig. 6A). In contrast, there was no appreciable difference in retinal cell death between GST-pro- and GST-injected (control) retinas (Fig. 6B) or between GST-injected and PBS injected retinas (PBS: 100.00±7.83 vs. GST: 107.53±4.05; O.D./µg protein (mean) ± SEM, n = 3, non-significant, Student's t test). We therefore concluded that GST-pro delivery into the mouse retina represents a safe procedure to block sortilin function in vivo and it was selected to test the effect of sortilin in intense light-induced retinal cell death in vivo.

Albino mice were subjected to intense illumination for 7 h, and their experimental and contralateral eyes were then injected with 1 µl of 1.5 µM GST-pro and 1 µl of 1.5 µM GST, respectively. After 24 h in darkness, the mice were sacrificed and the amount of nucleosomes was quantified in retinal cytosolic extracts, revealing significantly reduced cell death in the experimental retina in comparison to the control retina (Fig. 6B). Given that retinal cell death in albino mice subjected to intense illumination is restricted to the photoreceptor cell layer [31], these results demonstrate that sortilin participates in the photoreceptor degeneration in this model system and that its inhibition can reduce photic injury-derived cell death in vivo.

## Discussion

In this study, we show that intense lighting induces the expression of sortilin, at both mRNA and protein levels, in cells located within the ONL of albino mouse retinas, at the same time that p75^NTR^ and proNGF are expressed by these cells. A similar situation is observed in 661W cells (this study; [27]). We also show that sortilin is involved in light-induced photoreceptor degeneration, finding that neurotensin and the pro-peptide, two known sortilin ligands that compete with proNGF and prevent proNGF-induced apoptosis, significantly reduce the death of 661W cells subjected to intense light. In vivo, the pro-peptide significantly diminished cell death in the retina of albino mice subjected to intense illumination. Proneurotrophins other than proNGF could also participate in light-induced photoreceptor degeneration. Actually, proBDNF and proNT3 may both trigger sortilin/p75^NTR^-proapoptotic signals in a number of cell systems [36], [37].

We found that *Ngfr*-specific transcripts encoding the p75^NTR^ protein can be detected in the retina, delineating most cell somas present within the ONL, in both control and light-exposed albino mice. This observation, along with the observed expression of p75^NTR^ in 661W cells subjected to lighting (this study, [27]), demonstrates that photoreceptors can express p75^NTR^, as observed for other retinal cell types, such as Müller glial cells [25], [38].

The retina of albino mice expresses p75^NTR^ before being subjected to intense light, unlike findings for non-albino rodents [26], [27]. This suggests that albino retinas are already stressed under normal levels of light, consistent with evidence that p75^NTR^ is a stress-responsive receptor [39]. Albino mouse retinas therefore appear to be susceptible to degeneration even at low levels of lighting, a situation resembling the increased light-sensitivity observed in RP patients, in which light restriction might benefit the course of disease [40].

Not only p75^NTR^ but also sortilin showed increased expression in photoreceptor cells at 6 h post-illumination, in agreement with its capacity to be expressed in response to stress in the nervous system [14], [19], [20]. As noted above, sortilin remains detectable by immunohistochemistry in the ONL at 24 h post-illumination, despite the scarce expression of the corresponding gene. This suggests that the protein has a relatively long half-life, which would explain the observation of the presence of sortilin in the GCL of control retinas despite the absence of detectable *Sort1* mRNA expression in this retinal layer.

Enhanced sortilin and p75^NTR^ protein expression in rat retina was also reported after elevated intraocular pressure-induced retinal ischemia leading to generalized retinal cell death [41]. However, these proteins were mainly expressed by Müller glial cells in that situation [38], indicating that the p75^NTR^/sortilin receptor complex can be expressed by different retinal cell types according to the cause of cell degeneration and the neuronal phenotypes affected, resulting in diverse cellular mechanisms of apoptosis [42].

Sortilin was found to facilitate signaling through the gp130/Leukemia Inhibitor Factor Receptor β heterodimer [43], which is known to enhance the survival of photoreptors in albino mice subjected to photic injury [44], [45]. The relatively modest effect triggered by the pro-peptide in our in vivo experiments may be explained by the opposed signaling pathways in which sortilin appears to participate (i.e., death in response to proNGF and survival in response gp130 activation). Nevertheless, sortilin inhibition with the pro-peptide has a net positive effect. Further studies are required to enhance the effect observed with the sortilin blockers both in vitro and in vivo.

Sortilin activation is able to induce TNFα expression in microglial cells [46], and TNFα participates in photoreceptor cell death in two different animal models of RP [47], likely due to the capacity of resident retinal glial cells to produce this cytokine [48]. 661W cells can also express TNFα in response to medium conditioned by activated microglial cells [49]. This suggests that sortilin-dependent TNFα production might participate, along with the sortilin/p75^NTR^-specific signaling, in proNGF-dependent apoptosis. The TNFα/sortilin pathway might be exacerbated in the absence of p75^NTR^, thus explaining the lack of protection to photoreceptor degeneration observed in *Ngfr* ^−/−^, but not in *Ngfr* ^+/−^, albino mice subjected to constant illumination [29].

Interestingly, the enhanced post-illumination expression of *Sort1*, *Ngf*, and, to a lesser degree, *Ngfr*, in the ONL was in the form of patches, indicating that luminic stress does not appear to affect all photoreceptors in the same manner. This is consistent with the observation that photoreceptor degeneration is not homogeneous throughout the retina [31], supporting the two stage model for the genesis of photoreceptor dystrophies [50].

It is noteworthy that, under control conditions, photoreceptors and 661W cells were both observed to produce proNGF, although in a lesser amount than after intense light treatment. Therefore, proNGF does not induce the cell death of photoreceptors in the absence of luminic stress but may rather have a neurotrophic effect, as described in other systems [51]. The observed increase in sortilin and/or p75^NTR^ likely favors proNGF-dependent cell death in photoreceptors.

**Figure 7:**
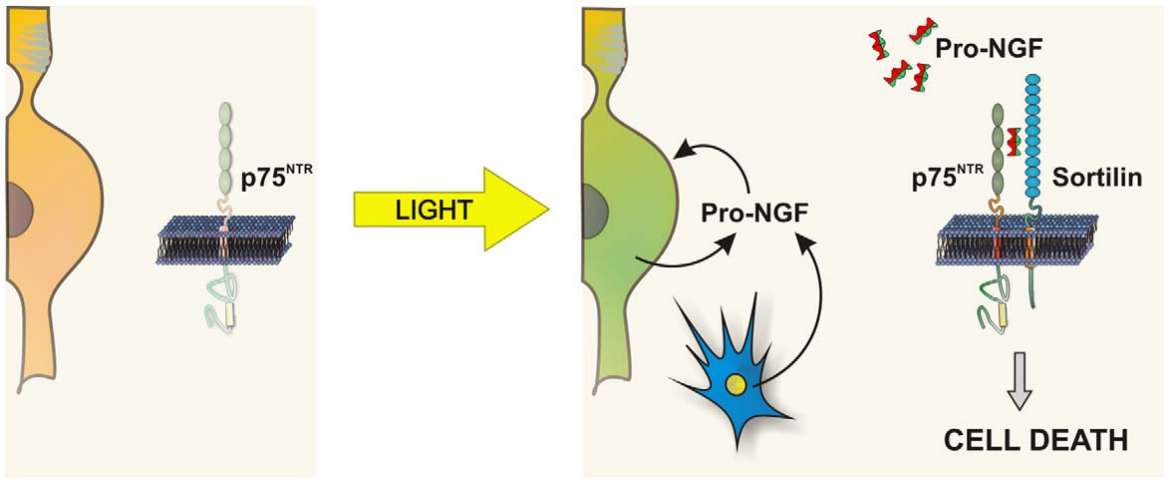
Scheme showing the proposed auto/paracrine mechanism regulating photoreceptor cell death in response to intense light. In the non-illuminated retina (left) photoreceptors express low levels of p75^NTR^ and undetectable levels of sortilin. Upon intense light exposure (yellow arrow) photoreceptors express high levels of sortilin and p75^NTR^ and they release proNGF into the extracellular milieu, an effect that can be potentiated by the release of proNGF from other sources such as activated microglial cells (blue cell). The binding of proNGF to the p75^NTR^/sortilin co-receptor complex (right) would lead to photoreceptor cell death.

In conclusion, our results point to an auto/paracrine mechanism in the retina that favors the apoptotic response of photoreceptors subjected to intense lighting (Fig. 7). The first stage of this mechanism is the induction in the photoreceptors of sortilin and p75^NTR^ expression and the release into the extracellular milieu of proNGF produced by the photoreceptors themselves, although it may be potentiated by proNGF from other sources such as microglial cells [30], [52] and/or by changes in growth factor expression [25]. The subsequent binding of proNGF to the p75^NTR^ and sortilin receptors would favor the death of photoreceptors. A similar mechanism was also observed in 661W cells, which release proNGF into the culture medium at the same time as light treatment enhances the expression of p75^NTR^ and sortilin proteins. Inhibition of sortilin function in 661W cells and in vivo was able to significantly rescue photoreceptors from intense light-induced apoptosis, further supporting our hypothesis. We therefore conclude that sortilin represents a putative target for intervention in hereditary retinal dystrophies.

## Materials and Methods

### Ethics Statement

Experimental procedures were approved by the animal experimentation ethics committee of the University of Granada (Permit Number: 2011-357), following the guidelines of the European Union Directive 2010/63/EU on the protection of animals used for scientific purposes.

### Mice

Adult female BALB/c albino mice (Harlan, Barcelona, Spain) of 60 days postnatal age were used in this study. They were maintained in the animal house facility of the Cajal Institute under a normal 12 h light/dark cycle before the start of experiments. Mice were sacrificed by cervical dislocation.

### Primary Antibodies

The mouse mAb 27/21 recognizing the native form of NGF [33] was used for coating ELISA plate microwells at a concentration of 440 ng/ml. Rabbit polyclonal antiserum [9992] against the intracellular domain of human p75^NTR^, a kind gift from Moses V. Chao (New York University), was diluted 1/1,000 for immunohistochemistry. Biotinylated goat anti-mouse sortilin antibody (R&D Systems, Minneapolis, MN) was used at 1/700 dilution for immunohistochemistry [14] and 1/5,000 dilution for western blot. Rabbit antibody against the pro-peptide, previously described by [32], was a generous gift from Carme Espinet (University of Lleida, Spain). This antibody was obtained by immunizing rabbits with a GST-fusion protein containing the Asp23-Arg81 peptide from human pro-NGF. Then, specific antiserum was purified by first incubating whole serum with GST to adsorb GST-specific immunoreactivity, followed by adsorption to, and elution from, a glutathione column to which GST-pro-NGF was immobilized. The antibody was observed to be specific as the immunoreactivity was abolished by the immunogenic peptide [32]. This antibody was used at 1/500 dilution for immunohistochemistry, 1/1,000 dilution for ELISA, and 1/10,000 dilution for western blot. Mouse monoclonal antibody against neuron-specific βIII tubulin (clone 5G8; Millipore, Billerica, MA) was used in western blot at 1/20,000 dilution.

### Plasmids

pGEX4T-propepNGFβ plasmid, which expresses the pro-peptide (amino acids E19 to R121) as a GST-pro fusion protein [11] was kindly provided by Anders Nykjaek (Aarhus University). pGEXKG plasmid, kindly provided by Mariano Carrión-Vázquez (Cajal Institute), was used to express recombinant GST.

### Recombinant Proteins

GST-pro and GST were expressed in *Escherichia coli* and purified on glutathione agarose beads (GSTrap FF columns, GE Healthcare), as previously described by [11].

### Cell Culture

661W cells [53] were a kind gift from Dr. Muayyad Al-Ubaidi (University of Oklahoma Health Sciences Center, Oklahoma City, OK). They were obtained from transgenic mouse retinas expressing SV40 T antigen and found to maintain photoreceptor phenotypes [53], [54]. These cells express a number of cone-specific markers such as cone blue and green opsins, transducin, and cone arrestin, but not of rod-specific proteins (rod opsin and rod arrestin), suggesting 661W cells arise from a cone photoreceptor lineage [55]. 661W cells were maintained in Dulbecco's Modified Eagle's Medium containing GlutaMAX-I and 4.5 mg/ml glucose (Invitrogen), plus 50 U/ml penicillin/streptomycin (Invitrogen) and 10% fetal bovine serum (Invitrogen).

### Induction of Light Damage

Photic injury to albino mouse retinas was carried out as described by [31] with some modifications. Briefly, animals were dark-adapted for 24 h before exposure for 7 h to cool white light (Master PL Electronic 23 W, 230–240 V Cool Daylight, Royal Philips Electronics, Amsterdam, Holland) at a luminescence level of 10,000 lux. The mice were then kept in complete darkness for 6, 12, or 24 h.

Light damage in 661W cells was induced as previously described [27]. Briefly, cells were grown to 80% confluency in growth medium and then transferred to serum free medium, where they were cultured for 18 h before exposure for 3 h to cool white light (Master PL Electronic 23 W, 230–240 V Cool Daylight) at a luminescence of 15,000 lux. Cultures were then returned to the incubator for a further 2 h (for RT-PCR), 3 h (for western blot), or 24 h (for ELISA and cell survival). Cells maintained under similar conditions but not exposed to illumination served as controls. This procedure has been shown to induce p75^NTR^ expression in light-damaged 661W cells [27].

### Histology and Immunohistochemistry

Entire enucleated eyes from control and light-exposed animals (three mice for each experimental point) were fixed in periodate lysine paraformaldehyde [56] for 6 h at 4°C. The fixed material was cryoprotected in PBS containing 30% sucrose, soaked in OCT compound (Sakura Finitek Europe, Zoeterwoude, The Netherlands), and frozen in liquid nitrogen. Blocks were stored at −40°C until use. Transverse sections (20 µm) were obtained in a cryostat (Leica, Wetzlar, Germany) and collected on SuperFrost slides (Menzel-Glässer, Braunschweig, Germany).

Cryosections were permeabilized and blocked for 30 min at room temperature in PBS containing 0.5% Triton X-100 (Sigma, St. Louis, MO) and 10% fetal calf serum (FCS; Invitrogen, Paisley, UK), and then incubated over night at 4°C with the primary antibody diluted in PBS/0.1% Triton X-100 plus 1% FCS. After 5 washes with PBS/0.1% Triton X-100, the sections were incubated for 1 h at room temperature with Cy2 conjugated anti-rabbit IgG (H+L) antibody (Jackson Immunoresearch, Newmarket, UK) or Alexa Fluor 594 donkey anti-goat IgG (H+L) antibody (Invitrogen), each diluted 1/1,000. Sections were finally washed 5 times in PBS/0.1% Triton X-100 and mounted in glycerol/PBS (1∶1).

### Tissue and Cell Extracts

Retinas were dissected out from the pigment epithelium, homogenized in Laemmli's buffer, and boiled for 5 min. 661W cell cultures were placed on ice, washed with ice cold PBS, and incubated for 30 min with 700 µl lysis buffer containing 50 mM Tris-HCl pH 8.0, 150 mM NaCl, 1% Triton X-100, and 1× protease inhibitor cocktail (Roche Diagnostics, Mannheim, Germany). Cell lysates were scraped with a rubber policeman and centrifuged at 13,000×g for 10 min at 4°C. Supernatants were 10 fold concentrated with centrifugal filter units (cut off: 3 kDa; Millipore), mixed with 1 volume of 2× Laemli's buffer, and boiled for 5 min.

### Western Blot

Cell or tissue extracts obtained as described in the Tissue and Cell Extracts section were separated by sodium dodecyl sulfate polyacrylamide gel electrophoresis on 13% polyacrylamide gels and transferred to Immun-Blot PVDF membranes (BioRad, Hercules, CA). Membranes were incubated for 1 h with 2% enhanced chemiluminescence (ECL) Advance blocking agent (ECL Advanced Western Blotting Detection Kit; GE Healthcare, Munich, Germany) in PBS plus 0.1% Tween 20 (PBT), and incubated for 2 h at room temperature with the appropriate antisera in blocking buffer. The membranes were washed five times in PBT, and then they were incubated for 1 h at room temperature with a 1/1,660,000 dilution peroxidase AffiniPure goat anti-rabbit IgG (H+L) antibody (Jackson Immunoresearch), or 1/500,000 peroxidase-conjugated AffiniPure donkey anti-goat IgG (H+L) antibody (Jackson Immunoresearch) in blocking buffer. Finally, they were washed again as above, and the protein bands were visualized using ECL Advanced Western Blotting Detection Kit (GE Healthcare).

### Cell Death/Survival Quantification

An ELISA, using a combination of antibodies recognizing histones and DNA (Roche Diagnostics), was used to quantify cell death in the retina in vivo. This method, previously described by [4], quantifies cell death as the level of soluble nucleosomes present in cytosolic retinal extracts. Briefly, retinas were homogenized in 200 µl containing 1× protease inhibitor (Roche Diagnostics) and centrifuged at 20,000×g for 10 min. A portion of supernatant was used to quantify proteins by standard methods (BioRad Protein Assay), and the rest was diluted 1/15 (illuminated) or 1/10 (darkness) in the supplied buffer and processed as indicated by the manufacturer. Results are shown as optical density (OD) per unit of protein (µg) present in the extract.

To quantify cell survival in vitro, 661W cell cultures were fixed with 4% paraformaldehyde (PFA) (Merck, Darmstadt, Germany) for 15 min at room temperature, and the nuclei were stained with 1 µg/ml bisbenzimide (Sigma). The degree of cell survival was determined by counting the number of non-pyknotic nuclei in the cultures. Cells were counted by using a Leica DMI6000 B inverted microscope (Leica) with phase contrast and epifluorescence illumination. Randomly taken pictures were taken with a Leica DFC350 FX digital camera (Leica), and subsequently analyzed with ImageJ (NIH, Bethesda, MD) software using the particle analysis (nucleus counter) plugin. On average, 8,000 nuclei were analyzed per experimental point.

### In Situ Hybridization

Digoxigenin labeled antisense riboprobes were synthesized as described previously [57]. Partial sequences from the coding region of *p75ntr* (currently known as *Ngfr*, bp 2,158–2,842; accession number NM_033217), *Sort1* (bp 5,792–6,411; accession number NM_019972), and *Ngf* (bp 336–846; accession number NM_013609) were obtained by RT-PCR using mRNA derived from E15 mouse embryos (Quick-prep Micro mRNA Purification Kit; GE Healthcare) and converted to cDNA with the First strand cDNA synthesis kit (GE Healthcare). The cDNA fragments were then cloned into the pGEM-T Easy vector (Promega, Madison, WI). Digoxigenin-labeled antisense riboprobes were obtained from linearized plasmid templates using T7 or Sp6 RNA polymerases as appropriate (Promega), following the manufacturer's instructions. In situ hybridization was performed at 60°C in 12 µm cryosections from different three mice for each experimental point following a previously described protocol [57]. For each riboprobe, cryosections were processed in parallel and incubated with substrate for identical time periods.

### RT-PCR

mRNA from 661W cells was extracted by using the QuickPrep Micro mRNA purification kit (GE Healthcare), from which cDNA was prepared using the First-strand cDNA synthesis kit (GE Healthcare). PCR amplification was performed using standard protocols with the following oligonucleotides: *βActin* (bp 163–182 and complementary to bp 300–321, accession number NM_007393), *Ngfr* (bp 2,158–2,177 and complementary to bp 2,823–2,842; accession number NM_033217), mouse *Sort1* (bp 5,792–5,811 and complementary to bp 6,392–6,411; accession number NM_019972), and *Ngf* (bp 336–355 and complementary to bp 827–846; accession number NM_013609). *βActin*, *Sort1*, and *Ngf* were amplified for 25–28 cycles, whereas *Ngfr* was amplified for 34–37 cycles. Under these conditions, amplification was linear.

### ELISA

The presence of soluble proNGF in conditioned media was detected by employing a modification of the ELISA protocol described by [33]. Briefly, ELISA plate microwells were incubated overnight at 4°C with 25 µl of anti-NGF mAb 27/21 (440 ng/ml in PBS). Microwells were then washed twice with PBS, and the remaining sites for protein binding were blocked for 2 h with 100 µl of 3% bovine serum albumin (Sigma) in PBS (PBSB). After two additional washes with PBS, 90 µl of conditioned media were incubated for 2 h in triplicate. Microwells were then washed four times with PBS, followed by a 2 h incubation with 50 µl rabbit anti-proNGF antibody (1/1,000 dilution in PBSB). The presence of this latter antibody was revealed with anti-rabbit horseradish peroxidase (1/5,000 dilution in PBSB), followed by incubation with 2,2′-azino-di-[3-ethyl-bezothiazoline-6 sulfonic acid] (ABTS; Roche Diagnostics) according to the manufacturer's instructions. The OD was then obtained at 405 nm.

### Blockage of ProNGF Signaling

Neurotensin is a known sortilin ligand [58], [59] that inhibits the binding of proNGF to sortilin and therefore interferes with the apoptotic effect of proNGF [11], [20]. Blockade of sortilin-dependent apoptosis can also be achieved by using the GST-pro fusion protein [11]. GST-pro has been shown to prevent the binding of proNGF to sortilin [11], consequently blocking the death signaling of proNGF [11], [34].

Neurotensin or GST-pro was injected into one eye of illuminated or control mice to prevent proNGF signaling in vivo. Eye injections were performed following the method described by [60]. Briefly, mice were anesthetized with isoflurane. Then, 1 µl of solution containing 90 µM neurotensin (Sigma) in PBS or 1.5 µM GST-pro in PBS were injected just behind the limbus with a 33 gauge beveled needle (World Precision Instruments, Sarasota, FL) using a NanoFil microsyringe (World Precision Instruments). Contralateral eyes injected with 1 µl PBS or 1.5 µM GST in PBS were used as controls. The mice were then maintained in complete darkness for an additional 24 h period prior to analysis.

For in vitro experiments, 10 µM neurotensin (Sigma) or vehicle (PBS) was added to the cultures at the beginning of intense light treatments. Alternatively, GST-pro or GST (both at a final concentration of 100 nM) were added to the culture medium at the beginning of light treatment.
